# Demographic aging: Implications for mental health

**DOI:** 10.4103/0019-5545.33251

**Published:** 2007

**Authors:** T. S. Sathyanarayana Rao, K. S. Shaji

**Affiliations:** Department of Psychiatry, J.S.S. Medical College and Hospital, Ramanuja Road, Mysore - 570 004, Karnataka, India; * Department of Psychiatry, Medical College, Thrissur - 680 596, Kerala, India

The proportion of older people in the world population is on the increase. For older people, mental health conditions are an important cause of morbidity and premature mortality. Among neuropsychiatric disorders, dementia and major depression are the two leading contributors, accounting respectively for one quarter and one sixth of all disability-adjusted life years (DALYs) in this group.[1] Special healthcare needs of the aged will have to be addressed by healthcare systems across the world. Functional dependency is common among older people and many would need assistance in the activities of daily living. Long-term care has become one of the major problems facing an aging society.

The most striking effects of population aging are to be seen in the most rapidly developing regions such as China, India and Latin America. Due to the unprecedented pace of demographic aging, these societies will have comparatively little time to develop social and healthcare policies to deal with the healthcare needs of the elderly in their population. Most people with dementia live in developing countries, the prevalence rate being 60% in 2001 and predicted to increase to 71% by 2040. Rates of increase are not uniform—numbers are forecast to increase by 100% in developed countries between 2001 and 2040, but by more than 300% in India, China and the South Asian and Western Pacific regions.[2] With its devastating negative effects on careers and families, dementia will soon emerge as a major public health problem in most developing countries. The number of elderly people suffering from depression and other mental health problems will also increase.

## CURRENT SCENARIO

According to the 2001 census, India is home to more than 76 million people aged 60 years and over. This age group, currently only 7.4% of the population, is expected to grow dramatically in the next few decades. Analysis of the census data shows substantial variation in the rate of demographic aging across India: at present, 10.5% of Kerala's population is older than 60 years while in Dadra and Nagarhaveli, this proportion is only 4%. Regions with more favourable health indicators seem to be aging faster and the demand for specialist services will soon be evident in such places.

The lack of priority accorded to the healthcare needs of the elderly seems to perpetuate the low level of public awareness about mental health problems of old age. Dementia and other mental disorders of older people, remain hidden problems rarely brought to the attention of healthcare professionals and policy makers. Cholinesterase inhibitors are now available at relatively low prices in India. This might lead to better identification and management of cases of dementia in clinical practice. Similarly, the availability of newer antidepressants with better side effect profiles will probably improve the identification and management of geriatric depression by physicians and general practitioners. Service development will become easier when health professionals and the public are more sensitive to the mental health needs of the elderly. There are hardly any specialized services for older people in the government-run public healthcare services in India compare to other speciality.

General health services remain clinic-based and typically involve long waits in crowded clinics for brief consultations. The usual focus in these settings is on ‘treatable’ acute pathologies and not on long-term care. Old people find it difficult to get to these clinics as it involves travel and use of transport. Doctors continue to be a rare commodity in rural settings. Trained health workers play a pivotal role in providing outreach services in rural areas. However, they do not consider the care of older people their priority unlike maternal and childcare. They have no formal training in identifying and providing intervention for problems like dementia or depression in older people. To correct this situation, health workers could be trained to identify potential cases of dementia in the community[3] and support home-based care. A shortage of trained manpower and inadequate budgetary allocation for mental healthcare will seriously limit the development of specialized psychogeriatric services across the country.

Specialized services are possible only in general or teaching hospitals depending on the availability of trained manpower. Obviously, we need to provide services in the primary care setting to benefit more people. At present, the primary care physicians do not come across many cases of dementia and are not involved in dementia care.[4] However, many older people with delirium and depression seek the help of the primary care doctor. Unfortunately, our undergraduate medical education does not give due importance to the healthcare of older people. This is not specific to our country.

Doctors and those responsible for commissioning and shaping health services have failed to acknowledge the rapid aging of most societies.[5] The family is the major provider of long-term care for the elderly. This is especially so in developing countries like ours where the family is the only source of care for disabled or behaviourally disturbed elderly people. Institutional care is unavailable in most places and is costly. It would be wrong to presume that home-based care is without economic and other costs. More often than not, such care is associated with considerable emotional as well as financial burden.[4,6,7] Most often, the caregivers are women and have very little access to or control of the resources needed to assume this responsibility. The existing community outreach services are not equipped to support home-based care of older people. Societies are in transition. Due to various social, economic and demographic changes, family resources are dwindling. The family size is getting smaller. Migration is on the rise resulting in changes in rural and urban social environments. Poverty and disability in non-elderly family members can make the care of elderly individuals more difficult. Care of elderly people at home, especially those with disabling mental health conditions such as dementia, will become increasingly difficult in the future as younger women upon whom the duties of care most frequently fall, are increasingly likely to work outside their homes.

## CHALLENGES AHEAD

It is estimated that there are already about 1.5 million people affected by dementia in India and this number is likely to increase by 300% in the next four decades.[2] The number of people affected by depression and other mental health problems will be more than this. The next few decades will see a sudden unprecedented increase in the number of older people needing mental health interventions. Resource limitations will remain and continue to be a matter of concern. Rising healthcare costs will make out-of-pocket payments unaffordable. The absence of social security schemes and very limited pension coverage are other serious limitations.

Shortage of professionals catering to the healthcare of the elderly, especially of geriatricians, psychiatrists and neurologists will continue. The curriculum of undergraduate and postgraduate medical education needs to be modified to meet the needs of the aging population. Training and deployment of outreach services is another area, which requires immediate attention. Care of older people will have to be recognized as a priority by the outreach services. A clear-cut policy as well as political will is needed for this.

Research, which informs and guides service development, is important. It can help us to allocate our limited resources cost-effectively. At present, there is little mental health research in India on public health issues such as burden, cost-effectiveness, services and program implementation. Building and sustaining research capacity as well as funding research are unresolved issues needing attention.[8]

## THE WAY FORWARD

Advances in knowledge about diverse aspects of the mental health of older people need to be translated to user-friendly services. Future development of services for older people needs to be tailored to suit the context of health systems. “Health systems” here can be taken to include macroeconomic factors, social structures, cultural values and norms and existing health and welfare policy and provision.[9]

In low-income countries with limited resources, the focus must be on primary care. To begin with, the community-based primary care systems staffed by doctors, nurses and health workers need to be enabled to care for older people. This would necessitate a paradigm shift beyond the current preoccupation with simple curative interventions. Primary care needs to encompass long-term support and chronic disease management. Doctors in primary care should be able to identify and manage delirium, dementia and depression.

Clinicians need training in the diagnosis and management of common mental health problems of the elderly. This will have to be provided to them. Better knowledge and skill will enable them to monitor and guide the outreach services effectively. Their guidance and support alone can ensure the good quality of outreach services. The National Initiative on Care for Elderly (NICE) is a central government program, which aims to enhance the delivery of care to the elderly in the community setting. NICE had started two new training courses; one a six-month certificate course in geriatric care and the other a one year postgraduate diploma course in integrated geriatric care. These courses will have to be made available in all states immediately. Such courses can generate more trained manpower. Outreach services across the country need a large number of trained health workers to provide community care for older people. It is important to provide training in geriatric care to all health workers in the existing primary care system. A brief two-week training module should be developed for these health workers. All of them should undergo this as a mandatory in-service training program. Health workers in the private sector too need similar training. Such short courses will also be useful for people employed by families for homecare. Many families employ homecare workers for assistance. Most often, these are women with no training in geriatric or general health care.

The next level of care to be prioritized would be respite care, both in day centers and (for longer periods) in residential or nursing homes. Such facilities could act also as training resource centers for caregivers. Day-care and residential respite care are more expensive than home care. But these are needed to meet the community's needs, particularly for people with more advanced dementia. Private sector and nongovernmental agencies may be encouraged to establish such centers. Mechanisms for ensuring the quality of services would rest with the government. Cost of long-term care will have to be met. Free services integrated with primary care will help to reduce the cost of care. Government should take steps to make pensions and other social security benefits available to cover most people. Financial support to caregivers who are unable to go for their daily work due to more pressing needs of the elderly, needs serious consideration. Another priority is to develop Geriatric Psychiatry as a subspeciality. It is indeed gladding that a speciality centre for Geriatric Psychiatric care has already started functioning at Lucknow. Geriatric Psychiatry units need to be established in psychiatry departments in all the medical colleges.

Geriatric Psychiatry should be given due importance during postgraduate training in Psychiatry. We need to consider the possibility of starting specialization courses in Geriatric Psychiatry. Research is important for service development. Good quality research can help us to allocate resources cost-effectively. Researchers need to be supported and their networking encouraged.[10] Dissemination of knowledge is also important for improving the outcome of mental health conditions. The recent initiative by the task force of the Indian Psychiatric Society to develop and distribute clinical guidelines for common geriatric psychiatric problems is a step in this direction. Continued efforts at dissemination of research findings need to be regularly undertaken.
